# Augmented screwdrivers can increase the performance of orthopaedic surgeons compared with use of normal screwdrivers

**DOI:** 10.1038/s41598-022-24646-z

**Published:** 2022-11-22

**Authors:** James W. A. Fletcher, Verena Neumann, Juan Silva, Abigail Burdon, Karen Mys, Vasiliki C. Panagiotopoulou, Boyko Gueorguiev, R. Geoff Richards, Michael R. Whitehouse, Ezio Preatoni, Harinderjit S. Gill

**Affiliations:** 1 AO Research Institute Davos, Clavadelerstrasse 8, 7270 Davos Platz, Switzerland; 2 Department for Health, University of Bath, Bath, UK; 3 Department of Mathematical Sciences, University of Bath, Bath, UK; 4 Biomechanics Section, Department of Mechanical Engineering, KU Leuven, Leuven, Belgium; 5 Musculoskeletal Research Unit, Bristol Medical School 1St Floor Learning & Research Building, Translational Health Sciences, Southmead Hospital, Bristol, UK; 6 National Institute for Health Research Bristol Biomedical Research Centre, University Hospitals Bristol NHS Foundation Trust and University of Bristol, Bristol, UK; 7 Department of Mechanical Engineering, University of Bath, Bath, UK; 8 Centre for Therapeutic Intervention, University of Bath, Bath, UK

**Keywords:** Preclinical research, Biomedical engineering, Fracture repair

## Abstract

Orthopaedic screws insertion can be trivialised as a simple procedure, however it is frequently performed poorly. Limited work exists defining how well surgeons insert screws or whether augmented screwdrivers can aid surgeons to reduce stripping rates and optimise tightness. We aimed to establish the performance of surgeons inserting screws and whether this be improved with screwdriver augmentation. 302 orthopaedic surgeons tightened 10 non-locking screws to what they determined to be optimum tightness into artificial bone sheets. The confidence in the screw purchase was given (1–10). A further 10 screws were tightened, using an augmented screwdriver that indicated when a predetermined optimum tightness was reached. The tightness for unstripped insertions under normal conditions and with the augmented screwdriver were 81% (95% CI 79–82%)(n = 1275) and 70% (95% CI 69–72%)(n = 2577) (*p* < 0.001). The stripping rates were 58% (95% CI 54–61%) and 15% (95% CI 12–17%) respectively (*p* < 0.001). The confidences when using the normal and augmented screwdrivers respectively were 7.2 and 7.1 in unstripped insertions and 6.2 and 6.5 in stripped insertions. Performance improved with an augmented screwdriver, both in reduced stripping rates and greater accuracy in detecting stripping. Augmenting screwdrivers to indicate optimum tightness offer potentially enormous clinical benefits by improving screw fixation.

## Introduction

The majority of people will sustain a fracture in their lifetime, often requiring screw fixation to restore function and mobility^1^. Screws are the most commonly inserted orthopaedic implant, with millions inserted each year just in the UK, in a global market expected to reach a value of $1.96 billion by 2028^2^. Most screws are inserted manually, being tightened to a surgeon’s subjectively chosen amount. If screws are tightened too much, they strip the surrounding bone, reducing strength by > 90% and increasing fixation failure rates^3^. If failure occurs, treatment costs at least double alongside increased morbidity and mortality for patients^3^.

Data is limited on how well surgeons insert screws and is based on only 145 surgeons inserting a total of 1510 screws^4^, with most studies limited by having either one surgeon insert all screws, or many surgeons insert very few screws. However, these studies have shown that surgeons repeatedly perform poorly with more than one in every four screws inserted, stripping (irreparably damaging) the screw hole^4^. If representative of clinical practice, this would equate to millions of screws being poorly inserted each year. Currently there are no data to support how tight surgeons insert screws given the limitations and underpowering of previous studies, or data on how tight surgeons think they should be inserting screws.

Awareness of the torque applied during screw insertion improves surgical performance^5,6^, though no work has utilised augmented screwdrivers in an attempt to aid surgeons to reduce stripping rates and optimise tightness. What torque should be targeted for optimum fixation has until recently been unknown. Previously, we have shown optimum non-locking screw tightness in certain conditions to be between 70 and 80% of the maximum torque^5,6^. Knowing what tightness to target, and augmenting screwdrivers to indicate when the optimum tightness has been reached, offers the promise of greatly improving surgical performance when inserting screws, though their use has not been explored.

The aims of this study were to identify for a large sample of orthopaedic surgeons what tightness is achieved when inserting non-locking screws, how tight surgeons think screws should be, how confident surgeons are in their insertions, their accuracy in detecting screw hole stripping, how screw insertions change when using an augmented screwdriver that indicates when optimum tightness is reached and how training and experience impact on outcomes.

## Methods

The study protocol, procedures and questionnaires were developed and approved under local institutional ethical approval (AO Research Institute Davos), in agreement with the Declaration of Helsinki. All attending surgeons, both faculty and participants attending an international orthopaedic course were eligible for inclusion in the study. Surgeons were invited to participate and/or surgeons presented themselves for testing. All participants gave informed, written consent to participate and for their anonymised data to be analysed. Neither financial nor material incentives were offered for participation—surgeons were told they would receive individually their results if they participated. Having read the conditions related to the study, participants completed a questionnaire in English for demographic information, before being asked to read instructions for the screw insertions.

**Figure 1:**
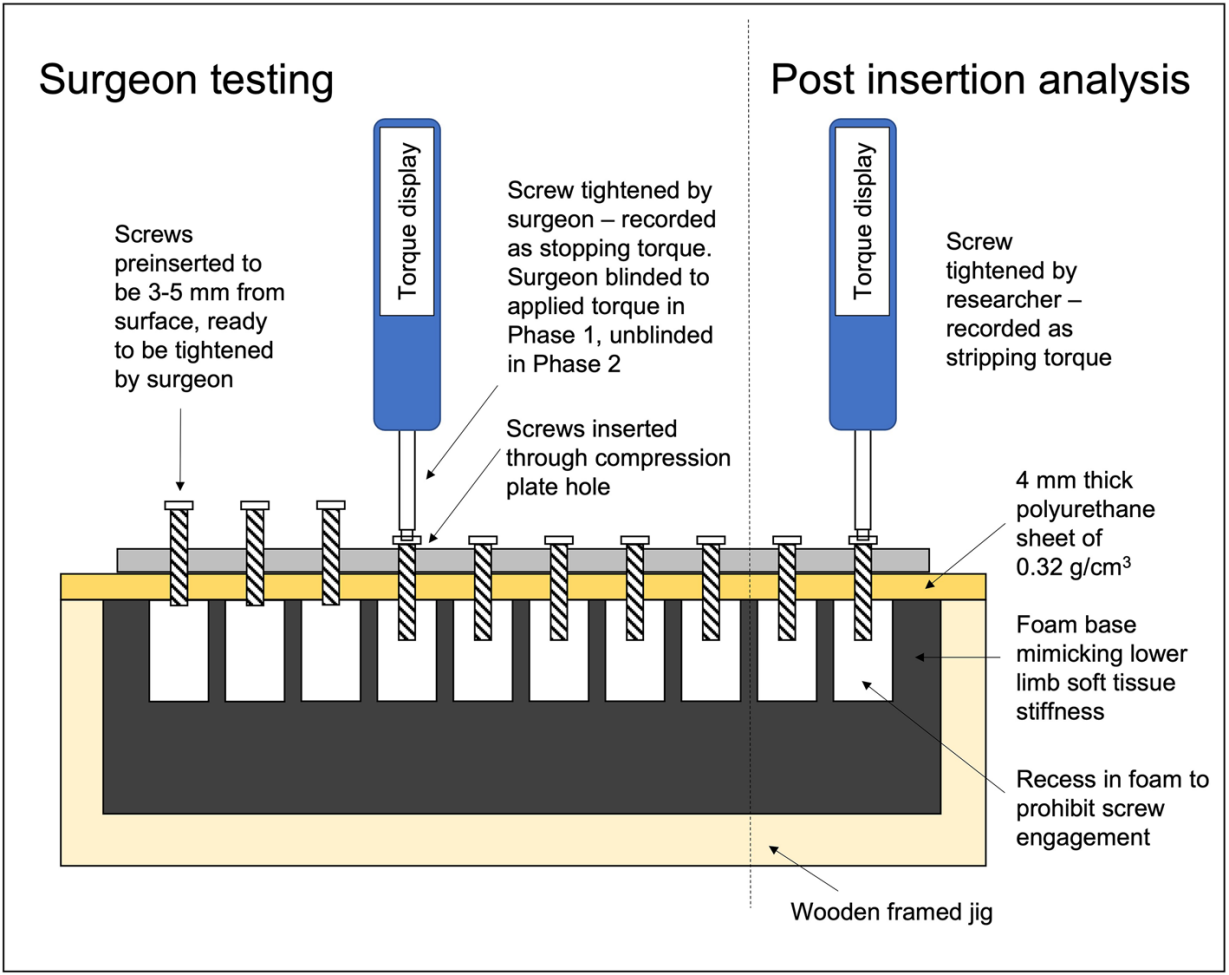
Diagram of testing arrangement showing screws pre-inserted to be 3–5 mm proud of the plate, to be tightened by the surgeon. Post insertion analysis performed at a separate episode.

Artificial bone sheets (Synbone, Zizers, Switzerland) of a density of 0.32 g/cm^3^ were made into sheets of 4 mm thickness using a custom-made milling process (FP1, Deckel Maho GmbH, Pfonten, Germany) with pilot holes of 2.5 mm diameter; these sheets were designed to mimic the difficult situation of performing insertion into low density bone (Fig. 1). Each sheet was mounted in a custom-made jig were using a base to mimic the stiffness of human lower limb tissue^7^.

**Figure 2:**
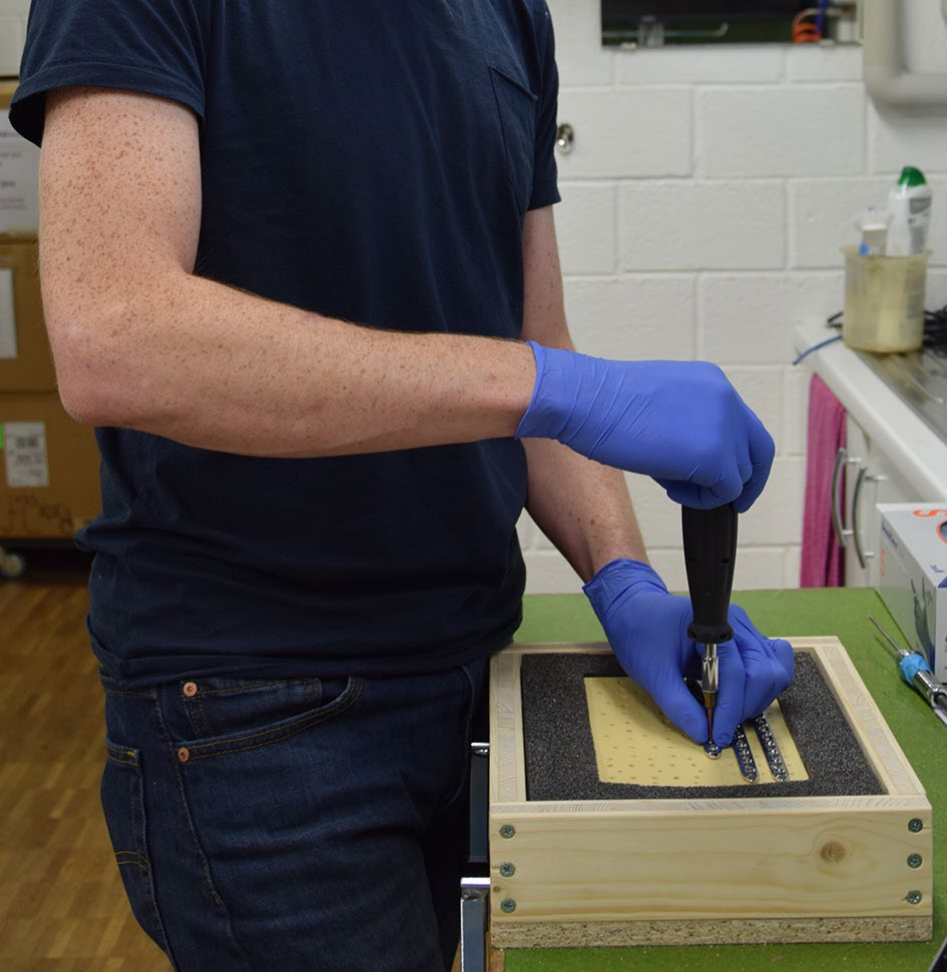
Demonstration of participant performing the screw insertion experimentation.

Surgeons were instructed to wear non-sterile gloves before sequentially tightening non-locking, 3.5 mm self-tapping, cortical screws into the artificial bone sheets in a vertical orientation in two testing phases (Fig. 2). All screws had been pre-inserted through 10-hole limited contact—dynamic compression plates (LC-DCP) (Synthes, Zuchwil, Switzerland), with the screws remaining 3 to 5 mm from the plate surface. In previous studies, we identified that no more than ten screw insertions are needed to characterise a surgeon’s technique^7,8^. A torque measuring screwdriver (Premier STS103 (Jack Sealey LTD., Bury St. Edmunds, UK)) was used for all screw tightening. Participants were asked to insert each screw to what they determined to be the optimum tightness for that screw. The screwdriver displayed the applied torque via a digital reading which was recorded by researchers; participants were blinded to these values. At a separate episode, a researcher calculated the surgeon’s achieved tightness by creating a ratio between the torque chosen by the surgeon (stopping torque) and the maximum torque the screw hole could receive (stripping torque). If the stopping torque was found to have been greater than the stripping torque, the insertion was defined as having been stripped by the surgeon. Following each screw insertion, participants rated the achieve purchase from 1 to 10 (1 being very poor and 10 being optimal). They also reported whether they felt the screw hole had been stripped—yes or no.

For Phase 2, 10 screws were tightened in exactly the same fashion except for the same screwdriver was set to beep and vibrate when a predetermined theoretical optimum torque value was achieved: 0.105 Nm. This value, defined as optimum tightness^5,6^, was calculated to be 70% of the average stripping torque for 3.5 mm screws in 2.5 mm screw holes in the 4 mm thick artificial bone sheets, established as 0.15 Nm from pilot testing. The instructions for inserting screws in Phase 2 were to stop inserting when the optimum tightness was indicated by the screwdriver. Again, confidence (1–10) in the screw purchase and the surgeon’s assessment of whether the screw hole had been stripped were recorded.

Statistical analysis was performed using paired t-tests for comparisons between phases of screw tightness, confidence in unstripped insertions, confidence in stripped insertions, sensitivity, specificity and accuracy in predicting screw hole stripping and prediction for optimum tightness and actual tightness achieved. Chi squared tests were used for stripping rate comparisons between phases. Regression analysis was performed using backwards stepwise regression to select from the following variables: age (< 26, 26–30, 31–35, 36–40, 41–45, 46–50, > 50), gender (female, male), job level (post-residency, pre-residency, residency), continent of work (Africa, Asia, Australasia, Europe, North America, South America), number of years in speciality, value thought to generate optimum tightness and engineering qualification (yes, no). Surgeons were categorised based on their techniques into either good performance (stripping rate ≤ 10% and an accuracy of ≥ 80%) or poor performance (stripping rate > 10% and/or accuracy < 80%) for both testing phases. Results were considered significant at a family wise error rate of 0.05 and confidence intervals were calculated at 95%. Statistical tests were performed with ‘R’, version 4.0.2^9^. All data are available in an online repository^10^.

## Results

Three hundred and two surgeons were recruited (Table 1). They tightened a total of 6040 screws, 3020 for each phase, with all screw insertions available for analysis.

**Table 1:** Demographic of participants.

	Number of surgeons (%)
**Age**	
< 26	6 (2.0)
26–30	40 (13.2)
31–35	55 (18.2)
36–40	58 (19.2)
41–45	50 (16.6)
46–50	36 (11.9)
> 50	57 (18.9)
**Continent of work**	
Europe	135 (44.7)
Asia	106 (35.1)
Australasia	11 (3.6)
Africa	18 (6.0)
North America	11 (3.6)
South America	21 (7.0)
**Gender**	
Male	276 (91.4)
Female	26 (8.6)
**Training**	
Pre-residency	14 (4.6)
Resident	58 (19.2)
Post-residency	230 (76.2)
**Engineering degree**	
Yes	25 (8.3)
No	277 (91.7)

**Figure 3:**
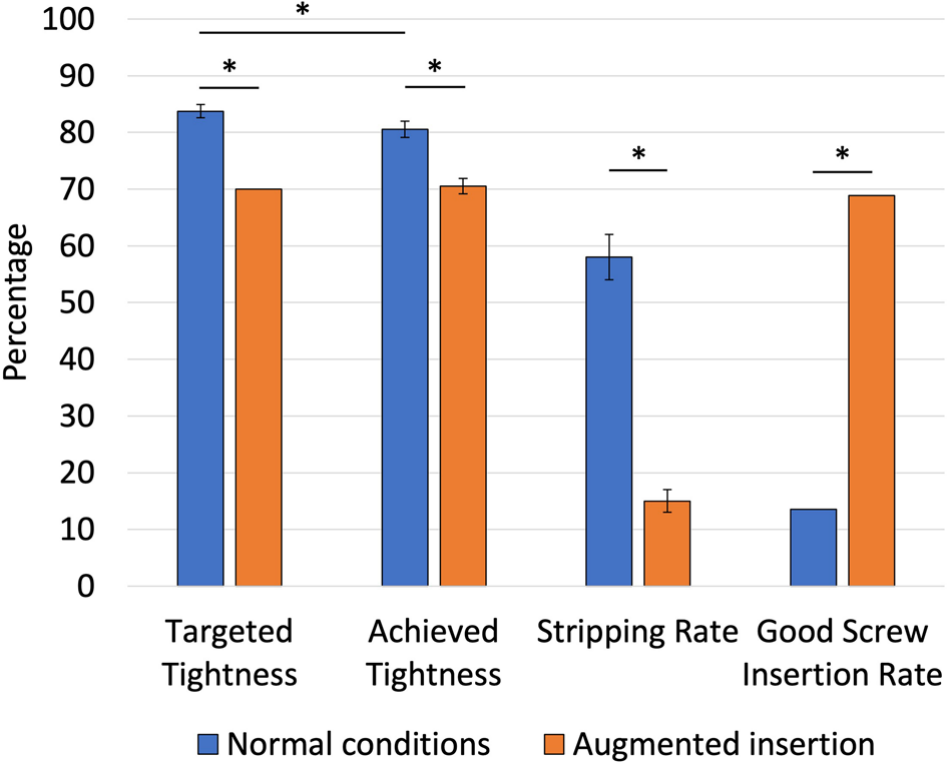
Surgeon performance for each of the testing conditions; normal screwdriver (blue bars) and augmented screwdriver (orange bars). Targeted tightness for normal insertion based on mean average reported by surgeons prior to insertion, and set to 70% when using the augmented screwdriver. Good performance defined as a screw stripping rate of ≤ 10% (i.e. no more than 1 of the 10 insertions were stripped) and an accuracy in correctly describing whether a screw hole was stripped or unstripped of ≥ 80% (i.e. at least 8 out of 10 answers about screw hole stripping were correct). Poor performance was defined as a failure to achieve one or both factors. Statistical differences (*p* < 0.001) highlighted with asterisk.

In Phase 1, using a normal screwdriver, 58% (95% Confidence Interval (CI) 54–62%)(n = 1745/3020) of screw holes were stripped, with the mean average screw tightness for unstripped insertions being 81% (95% CI 79–82%)(n = 1275)(Fig. 3). In Phase 2, with an augmented screwdriver, a lower stripping rate was seen of 15% (95% CI 12–17%) (n = 443/3020)(*p* < 0.001), with a lower mean average screw tightness for unstripped insertions of 70% (95% CI 69–72%)(n = 2577)(*p* < 0.001). In Phase 1, 56 surgeons (19%) stripped all 10 screw holes. This reduced to just seven surgeons (2%) in Phase 2 (*p* < 0.001).

Surgeons reported that the mean average tightness for optimum purchase should be 84% (95% CI 83–85%, range 50–100%), which was different to the average tightness achieved (81%) with a normal screwdriver (*p* < 0.0164) (Fig. 3).

**Figure 4:**
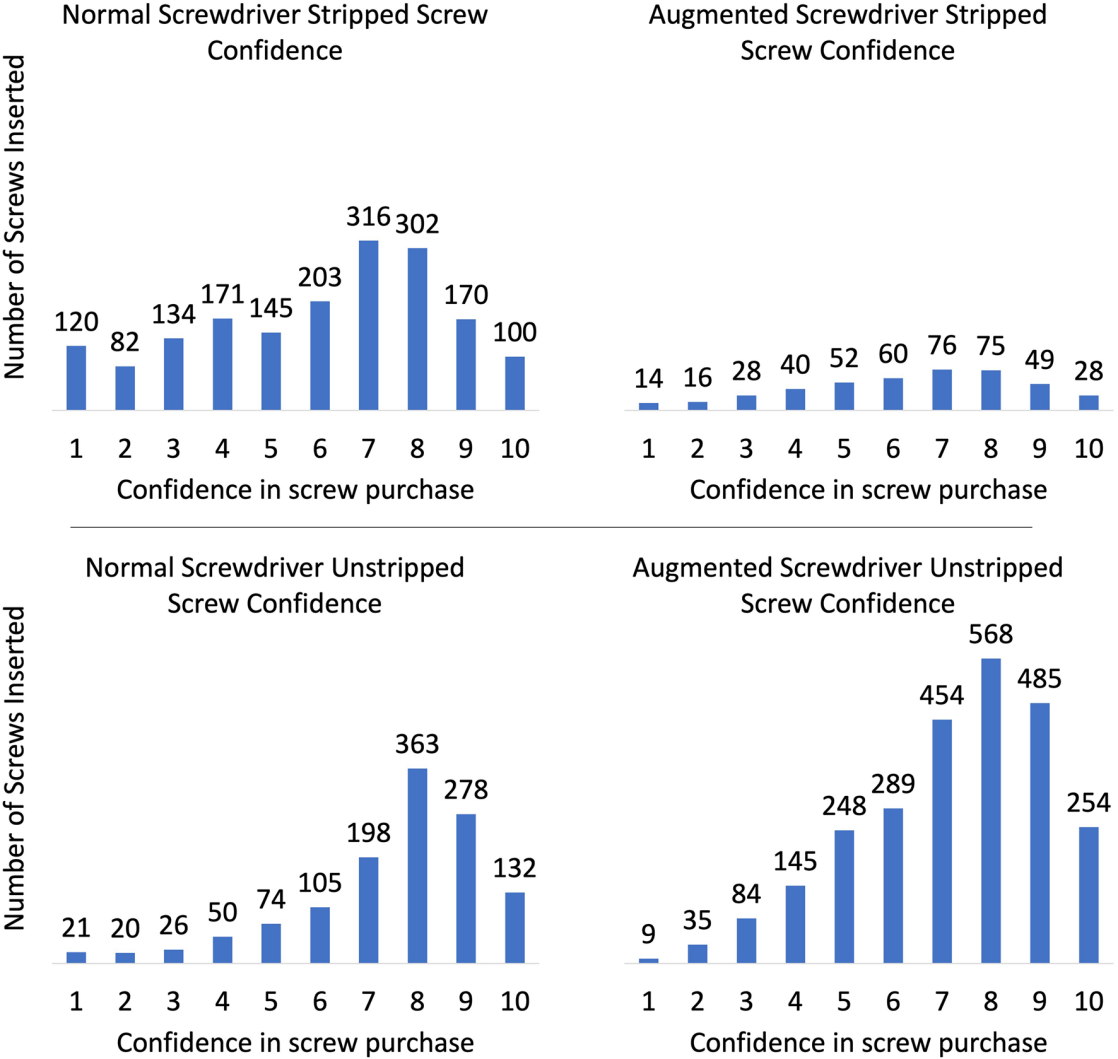
Confidence value reported for screw purchase for each testing condition (normal or augmented screwdriver) and when the screw hole was or was not stripped (1 being very poor confidence and 10 being optimal confidence).

Unstripped screw confidence did not change between phases: Phase 1—7.2 (95% CI 7.0–7.4), Phase 2—7.1 (95% CI 6.9–7.3) (*p* = 0.441) nor did confidence in stripped insertions: Phase 1—6.2 (95% CI 5.9–6.4), Phase 2—6.5 (95% CI 6.2–6.7) (*p* = 0.218) (Fig. 4). However, accuracy in detecting whether a screw had or had not stripped the hole on insertion increased significantly with the use of an augmented screwdriver: Phase 1—55%, Phase 2—85% (*p* < 0.001).

For Phase 1, no variables were seen to be associated with changes in screw tightness. Variables associated with a change in stripping rates were: job level (pre-residency 5.9% lower than post-residency and residency 13.6% lower than post-residency), predicted optimum (0.3% higher per value of prediction) and years of experience (0.4% higher per year of experience). For Phase 2, variables associated with overall average tightness were: having an engineering qualification (3.8% less tight), years of experience (0.1% tighter per year of experience) and continent of work (compared to reference category Africa, Asia 2.7% less tight, Australasia 6.7% less tight, Europe 6.7% less tight, North America 3.3% less tight and South America 1.3% less tight). Variables associated with a change in stripping rates were: years of experience (0.6% higher per year of experience) and continent of work (compared to reference category Africa, Asia 3.1% higher, Australasia 5.0% lower, Europe 6.4% lower, North America 10.9% lower and South America 0.4% lower). The three regression models with significant predictor variables were compared to an intercept only model which resulted in significant p-values of 0.0004, 0.0050 and 0.0001 respectively.

Using the categories based on stripping rate and accuracy in detecting stripping, using an augmented screwdriver led to good performance being seen in 69% of surgeons compared to just 9% under normal conditions (Fig. 3).

## Discussion

Surgical performance varied greatly amongst surgeons, with a considerable proportion of screws being inserted poorly. Use of an augmented screwdriver indicating when optimum tightness had been reached dramatically improved surgeons’ techniques. As stripped screw holes impact on bone healing^11^, fixation strength^5,6,12^ and contribute to fixations failing^3^, stripping rates should ideally be zero. An ability to immediately critique a screw insertion should enable the detection of stripping by the surgeon, should it occur, so that remedies can be enacted, such as changing the position of the fixation or increasing the screw size^12^. However, we found, as did Stoesz et al. previously, that detection of stripping when inserting screws with unaugmented devices is rare, and only when the bone is greatly damaged^13^. The high stripping rate seen with a normal screwdriver combined with the low accuracy in detecting stripping by some surgeons may indicate that sub-optimal fixations are being routinely performed. Equally, given the poor techniques seen with a normal screwdriver, it may be that current fixation strategies use more screws than are needed to compensate for some screws being inserted poorly. Using augmentation to indicate optimum tightness, the significant improvements in techniques seen could mean that fewer, better-inserted screws would provide the same fixation if they were inserted correctly. This could reduce surgical exposures, surgical time and implant costs.

Only a couple of the recorded demographic factors were associated with changes in technique, particularly number of years of experience and continent of work, implying that a spectrum of techniques is seen in all countries, ages and surgical experiences. Surgeons with higher values for the suspected optimum tightness were associated with higher stripping rates, perhaps due to a desire to achieve more tightness.

Whilst confidence did reduce significantly when screw holes were stripped, it still remained high in both normal and augmented insertions. Indeed, many surgeons reported mid-range and even high confidence in a screw that had clearly stripped the screw hole. This may reflect a general inability to critique insertions, or a lack of understanding by some surgeons of what proprioceptive feedback they should be feeling for. Perhaps even not knowing how a screw works. Thankfully, this improved with augmentation, demonstrating the safety benefits quantitative feedback can offer; in this study, techniques could be described as ‘good’ in 69% of surgeons when using augmentation, compared to just 9% under normal conditions. Unfortunately, it is not known, and we did not investigate, what a surgeon would do with a low confidence in a screw, i.e. at what confidence score would a surgeon change the screw or alter the fixation.

Previous work on screwdriver augmentation has shown its benefits^8,14^, but the tightness to target, and then how to target this have been unknown. Recently however, whilst only in-vitro biomechanical studies, optimum tightness for non-locking screws has been found between 70 and 80% of the stripping torque. Additionally, using bone characteristics^15,16^, the stripping torque can be calculated or at least estimated prior to insertion. This study demonstrates that assuming appropriate estimations of the optimum tightness can be made—which are straightforward in the controlled, artificial bone testing environment used—augmenting screwdrivers to indicate the optimum torque has great fixation benefits. With advancements in drill design, bone density estimates can be made based on the energy required to create the pilot hole^17^. This characteristic can be incorporated into the calculations for the maximum torque, and thus what 70 to 80% would be.

This is the largest study to date into surgeon performance when inserting screws. Most previous studies were underpowered due to the small number of screw insertions for each tested variable and/or a small number of surgeons^4^. It is the first study to look at different surgeon characteristics and whether these are associated with changes in performance and the first to look at augmented screwdriver insertion on a large sample. Furthermore, a diverse spectrum of surgeons was tested, increasing the generalisability of the findings.

Limitations with this study include that whilst having an international group for testing makes the findings more generalisable, there may have been language issues that made instructions harder to understand by some participants—though the courses they were attending were also being delivered in English. The homogeneity of the bone model used removed confounders from the testing material, though as it is artificial bone, its properties may differ from in vivo fixation techniques and outcomes. However, a previous study has shown that techniques in artificial bone mimic those in human bone^7^. The optimum tightness for this model was not investigated in this study and used previous investigations in bovine and human bone as a reference point for was tightness to target. The optimum tightness for artificial bone may be different, meaning that augmentation should have been set to a different level, however, the primary objective of screw insertion is to not stripping the surrounding bone, which augmentation was highly successful in achieving, whereas optimum tightness (which for this model is not known) is a secondary objective. Neither assessments of fixation strength nor of the impact on bone healing were performed, though stripped screws are known to worsen bone healing^11^. Finally, the benefits seen with the augmented screwdriver may have been enhanced due to greater familiarity with the material and stripping torque for Phase 2 compared to Phase 1. However, no difference has been seen in previous biomechanical studies between the first 10 insertions of a screw and more insertions^7,8^.

## Conclusions

Using augmentation reduces stripping rates, improves accuracy whilst optimising screw tightness. Developing methods for real-time evaluation of the stripping torque and use of torque-controlled screwdrivers will improve surgical performance through less failed insertions, saving time, money and likely improving patient outcomes.

## Data Availability

Data is available at the following online repository: https://doi.org/10.15125/BATH-00956.
